# Dorsal column pathway is involved in tactile reward-induced affective 50-kHz ultrasonic vocalizations in rats

**DOI:** 10.1371/journal.pone.0320645

**Published:** 2025-03-26

**Authors:** Rie Shimoju

**Affiliations:** Center for Basic Medical Research, International University of Health and Welfare, Otawara, Tochigi, Japan; University of Nebraska Medical Center College of Medicine, UNITED STATES OF AMERICA

## Abstract

Rhythmic stroking induces positive emotions in rats via the mesolimbic dopamine system. However, the ascending pathways underlying the affective 50-kHz ultrasonic vocalizations (USVs) induced by somatosensory stimulation remain unknown. The dorsal column consists of ascending spinal tracts that convey innocuous tactile information from the spinal cord to the brain. Here, we investigated whether the somatosensory signals transmitted through the dorsal column pathway contribute to the induction of positive 50-kHz USVs during rhythmic stroking. The 50-kHz USVs, behavior, approach latency, and mechanical tactile thresholds of animals with dorsal column lesions (DCL) at the upper thoracic level were compared with those in sham-operated animals. The DCL significantly reduced the number of 50-kHz USVs, harmonics, and split calls during rhythmic stroking, and the number of hedonic frequency-modulated calls (trill, complex, and step up calls) after rhythmic stroking. The DCL significantly increased the approach latency compared to presurgical controls. Sham-operated rats demonstrated a significant increase in the number of 50-kHz USVs and shortened approach latency compared with presurgical control values. Our results suggest that the somatosensory input conveyed by the dorsal column triggers the affective 50-kHz USVs during rhythmic stroking and approach behaviors. These findings contribute to revealing the neural circuits underlying somatosensory-emotional integration.

## Introduction

Rats are highly vocalizing mammals, and they engage in audible and ultrasonic vocalizations (USVs), which serve communicative functions and reflect emotional states [1,2]. Juvenile and adult rats emit specific USVs depending on their emotional state [3–5]. Rat USVs are mainly categorized into 22- and 50-kHz USVs based on their acoustic characteristics [6–8]. The 22-kHz USVs (frequency, 20–30 kHz; call duration, 300 to >  3000 ms), which are observed in negative aversive states, such as predator exposure, social defeat, acoustic startle, and foot shock [9–13], and typically accompanied by defensive submissive behaviors such as freezing and immobilization [14–16]. In contrast, the 50-kHz USVs (35–80 kHz, 10–150 ms), which have been proposed to reflect as a state of positive states, such as mating, food anticipation, psychostimulant drugs, rewarding electrical brain stimulation, and rough-and-tumble play [17–22], being typically accompanied by reward-related behaviors such as approach and locomotion [21,23–25]. The 50-kHz USVs can be divided into 14 different subtypes [26] associated with specific reward-seeking behaviors, such as play anticipation and approach [21,25,27]. In particular, frequency-modulated (FM) calls are considered indices of positive affective state, e.g., a state akin to human joy and laughter [4,20,28]. In addition, the number of 50-kHz USVs is negatively correlated with approach latency [21,28,29]. High rates of 50-kHz USVs and shortened approach latency have been described as indices of positive reinforcement and a highly motivated state [21,28,29]. Hence, rat USVs can be a useful tool for research on emotion and motivation and their underlying neural mechanisms [4,30–34].

We recently reported that rhythmic stroking (tactile stimulation with gentle movement) increases 50-kHz USVs via the mesolimbic dopamine system [27,28,35]. However, the ascending mechanisms involved in the emission of 50-kHz USVs induced by rhythmic stroking remain unknown.

Human and animal studies indicated that gentle stroking induces a positive affective state [36,37]. In human research, the degree of touch pleasantness varied depending on the speed and strength of stroking [38,39]. Recently, Köteles *et al*. reported that rhythmic stroking induces more pleasant sensations than rhythmic touching [40]. In addition, Case *et al.* found that when mechanoreceptive A-fiber function is greatly diminished, the perceived intensity and pleasantness of gentle stroking are nearly abolished [41]. In animal studies, we previously observed that rhythmic stroking can induce more hedonic 50-kHz USVs (especially FM calls, complex, and step up calls, and harmonics calls) than holding, light touch (tactile stimulation without movement), and swinging (mainly vestibular and proprioceptive stimulation) [27]. In addition, the brain theta activity associated with 50-kHz USVs, which are presumed to indicate a hedonic state, differed significantly between rhythmic stroking and light touch [42]. Further, rhythmic stroking induced a faster approach behavior than light touch [28]. Moreover, tickling (tactile stimulation with quick movement) induced a higher rate of 50-kHz USVs accompanied by accumbal dopamine release and faster approach behavior than light touch in juvenile rats [43]. These previous studies illustrated that light touch and proprioceptive stimulation are much less effective in inducing 50-kHz USVs than rhythmic stroking and tickling. Therefore, we hypothesized that innocuous mechanical receptors responsive to tactile stimulation with movement (discriminative touch) can be necessary for evoking hedonic 50-kHz USVs during somatosensory stimulation.

Several studies showed that somatosensory information transmitted from the spinal cord to the brain may be necessary for the emission of 50-kHz USVs during stimuli applied from rat’s body surface such as play behaviors. Play behaviors such as rough-and-tumble play and tickling are known to have a high rewarding valence and to evoke abundant 50-kHz USVs in juvenile rats [19–21]. Sivity and Panksepp reported that somatosensory input from the spinal cord sends excitatory projections to various brain areas during rough-and-tumble play [44]. Ishiyama *et al.* reported that the activation of the somatosensory cortex drives 50-kHz USVs during tickling [45]. However, Panksepp *et al*. reported that neonatal decortication and anesthetization of the dorsal region with xylocaine only partially affect play behavior [46,47]. Hence, affective play behavior accompanied by positive 50-kHz USVs exists even with reduced cutaneous somatosensory input. Given the drastic postural changes during play behavior, proprioceptive, vestibular, and visual changes may be also involved in the emission of 50-kHz USVs. Therefore, it is difficult to determine which stimulus modality is responsible for induction of affective 50-kHz USVs during play behavior. In contrast, rhythmic stroking is a suitable method for evaluating the effects of tactile stimulation on 50-kHz USVs, as it can be applied while the rat is in a relaxed state without drastic changes in the rat’s posture [27,48,49].

Two main ascending spinal pathways convey tactile information to the brain, being located in the dorsal and ventral funiculi on each side of the spinal cord [50]. The dorsal column tract is the major ascending pathway of innocuous somatosensory information including discriminatory touch. By contrast, the spinothalamic tract transmits crude touch and pressure information [50]. The dorsal column lesion (DCL) surgical procedure used in this study aimed to reduce the transfer of innocuous discriminatory touch information.

The present study aimed to investigate that the dorsal column pathways involved in the generation of positive emotion during rhythmic stroking. For this purpose, affective 50-kHz USVs and approach latency were evaluated before and after bilateral DCL at the upper thoracic level T2. In rodents, the dorsal column includes both sensory and motor tracts, therefore, we also evaluated behaviors and tactile thresholds to investigate motor and sensory deficits resulting from the lesion. We conclude that the somatosensory information conveyed by the dorsal column pathway are necessary to induce positive 50-kHz USVs.

## Materials and methods

### Animals

In total, 22 male Wistar/ST rats (7 weeks old, 220–260 g at the time of surgery) were used in this study (Japan SLC, Inc., Shizuoka, Japan). Five rats were used for preliminary experiments to examine surgical methods, and their recorded USVs were excluded from behavioral analysis. One animal died during surgery because of deep anesthesia, but no animal met the endpoint criteria during the recovery period after surgery. Therefore 16 rats were used (n =  8/group). Four animals were used per experiment, and each experiment lasted for two weeks, giving a total experimental duration of 8 weeks. After arrival, animals were housed in pairs in standard polycarbonate cages (W27 cm ×  L44 cm ×  H18 cm). The animals were maintained under controlled temperature (23 ±  1ºC) and light cycle (12-h/12-h light/dark, lights on at 08:00 h). Standard rodent food (Labo-MR stock, Nosan Corporation, Kanagawa) and water were provided *ad libitum*. After one week of acclimation to the laboratory environment, rats were individually housed and handled for 2 min daily for one week before surgery. Rats were randomly divided into two groups: sham DCL (sham, n =  8) and DCL (n =  8) (check methods for details about lesion surgery). USVs, behavior activities, approach latency, and tactile thresholds were measured in rats before surgery and on day 7 after surgery. All experiments were conducted between 9 a.m. and 4 p.m., in accordance with the Japanese Physiological Society’s Guide for the Care and Use of Laboratory Animals. The study protocol was approved by the animal ethics committee of the International University of Health and Welfare (Permission Number: 22006) and all efforts were made to minimize suffering and distress. All surgeries and handling were performed by a skilled experimenter.

### Dorsal column lesions

A bilateral DCL was performed at T2 careful to avoid damaging the descending dorsal corticospinal tract as much as possible. At this level of the dorsal column, the lesion was expected to deactivate the ascending afferent information in the stimulation area, including the trunk and lower body, except for the upper limbs, neck, and head [51]. Animals were anesthetized using sodium pentobarbital (40 mg/kg, i.p.) with isoflurane. The animal’s head was secured in a stereotaxic apparatus and the neck and thorax were gently fixed using a piece of cotton. The spinous process of T2 was used as an anatomical landmark to identify the other vertebrae [52]. A small sagittal incision was made to expose the T1–T3 vertebrae, a laminectomy was performed at T2 using a drill and bone cutter, and the dura mater incised to expose the spinal cord. First, a 27G needle was inserted at T2 (0.4 mm lateral to the midline on either side at a depth of 0.6 mm) [53]. Subsequently, the dorsal column was transected one side at a time using sharp microscissors without damaging the dorsal spinal vein. Finally, the medial dorsal column was crushed using a pair of fine forceps (No. 5 Dumont biology forceps) [54]. The fine forceps were inserted about 0.6 mm and the dorsal column and midline dorsal blood vessels compressed together. This procedure was repeated 4–5 times, causing an evident lesion in the medial dorsal aspect of the spinal cord, where dorsal column axons mainly carry information from Aβ low-threshold rapidly-adaptive mechanoreceptors [55]. Next, the exposed spinal cord was covered with a piece of gel foam and the muscles and skin sutured in layers using 6-0 and 3-0 silk, respectively. Sham-operated animals underwent only laminectomy at T2, without damaging the dura. A preventive antibiotic and analgesic therapy were administered before surgery (Carprofen 50 mg/kg, s.c.; Zoetis Japan Inc., Tokyo, Japan); after surgery, rats received a subcutaneous injection of 1% lidocaine (Sandoz AG, Tokyo, Japan) and an antibiotic (penicillin G procaine, 40,000 unit/kg, i.m.; Riken Vets Pharma Inc., Saitama, Japan) and were allowed to recover on a heating pad before returning to their cages. The rats were allowed a recovery period of 1 week, during which motor and sensory deficits were confirmed and no functional recovery was observed [56]. Animal health and behavior were monitored daily until the day of the experiment.

### Rhythmic stroking

Rhythmic stroking was conducted according to our previous study [27,35]. Briefly, each rat was gently held by the back skin and repeatedly (approx. 1–1.5 Hz) stroked on its ventral side for 30 s at a speed of 15–20 cm/s.

### Recording and analysis of ultrasonic vocalizations

We conducted the recording and analysis of USVs as previously described [28,35]. In brief, USVs were recorded using an UltraSoundGate 116H audio device with a CM16/CMA microphone (Avisoft Bioacoustics, Berlin, Germany). For acoustic analyses, the recordings were transferred to SASLab Pro (version 5.2, Avisoft Bioacoustics). 50-kHz USVs were analyzed manually according to Wright *et al*.’s classification [26] and our previous study [28,35].

### Behavioral recording and analysis

Before and after surgery, spontaneous behaviors were observed to detect any motor deficit caused by surgery. Behavioral recording and analysis were conducted as previously described [42]. In brief, behaviors were monitored using a digital video camera (DCR-PC120, SONY, Tokyo, Japan) while USVs were recorded. After the experiment, behavioral activities were manually scored offline by counting the number of occurrences and measuring the duration of the target behaviors. The total count (for rearing) or total duration (locomotion and exploring; standing against the wall and sniffing) were measured as behavioral parameters after rhythmic stroking.

### Approach latency

To examine an index of positive reinforcing/incentive value for rhythmic stroking, we measured the approach latency according to previous studies [21,24,28,57]. After recording USVs for the first 30 s stimulation, the rats received a second 30 s stimulation, after which they were immediately placed in the corner of the home cage. Approach latency was the time from this moment until the rats approached and touched the experimenter’s hand. The maximum latency was set to 30 s.

### von Frey tests

To detect sensory deficits due to surgery, mechanical sensory thresholds were measured in two separate regions, the lower mid-abdomen and the plantar surface of the right hindpaw. Animals were placed in an elevated polycarbonate test chamber (W13.5 ×  D21 ×  H13.5 cm) with a steel mesh bottom floor and habituated for 5 min before testing. Tactile sensitivity was measured using the up/down method with eight von Frey monofilaments (Neuroscience Inc.; Tokyo, Japan) (0.4, 0.6, 1.0, 2.0, 4.0, 6.0, 8.0, 15.0 g for the hindpaw; 2.0, 4.0, 6.0, 8.0, 15, 26, 60, 100 g for the abdomen) [58]. If an animal did not respond to the strongest von Frey monofilament (15.0 g for the hindpaw, 100 g for the abdomen), the 50% mechanical paw withdrawal response for that paw was calculated as 25.368 g for the hindpaw and 226.84 g for the abdomen according to a previous study [59].

### Experimental protocols

All measurements for each animal were made on the day of surgery and 7 days thereafter for both sham and DCL groups. In all experiments, we simultaneously recorded USVs and behavioral activity continuously for 90 s (30 s baseline, 30 s stimulation, and 30 s poststimulus) for each stimulation period. USV data were assessed before (30-s) (baseline), during (30-s), and after (30-s) rhythmic stroking. Behavioral data were assessed after stimulation. The von Frey test was performed after USV recording.

### Histology

After completing the experiments, all rats were deeply anesthetized with sodium pentobarbital (i.p., 100 mg/kg) and intracardially perfused with heparinized saline followed by 4% formaldehyde in 0.1 M phosphate-buffered saline. The thoracic spinal cord was removed and cryoprotected by placing it in a solution of 30% sucrose. The spinal cord was cut into 50-μm-thick sections using a cryostat (Leica CM3050 S; Leica Biosystems, Nussloch, Germany). Sections were stained with cresyl violet and examined microscopically to determine the location of the DCL by referring to the atlas of Sengul *et al*. [53]. Only rats for which the DCL at T2 was confirmed were used for analysis.

### Statistical analysis

Data are expressed as mean ±  SEM. The Shapiro–Wilk test was used to check the normality of datasets. Normally distributed data were subjected to parametric tests (three-way ANOVA followed by post hoc Bonferroni tests for the influence of the group (DCL or sham) ×  treatment (pre- or postsurgery) ×  time-course (before, during, and after stimulation) measurements, two-way ANOVA followed by post hoc Bonferroni tests for the influence of the group ×  time-course measurements and one-way ANOVA followed by post hoc Bonferroni tests for time-course measurements), and paired and unpaired t-tests for group comparisons. Non-normally distributed data were subjected to nonparametric tests (Wilcoxon signed rank test or Mann–Whitney U test for group comparisons). All statistical analyses were performed using SPSS ver. 23 (IBM Cor., Armonk, NY, USA). In all analyses, p <  0.05 (two-tailed) was considered to indicate statistical significance.

## Results

### Histology

**Fig 1:**
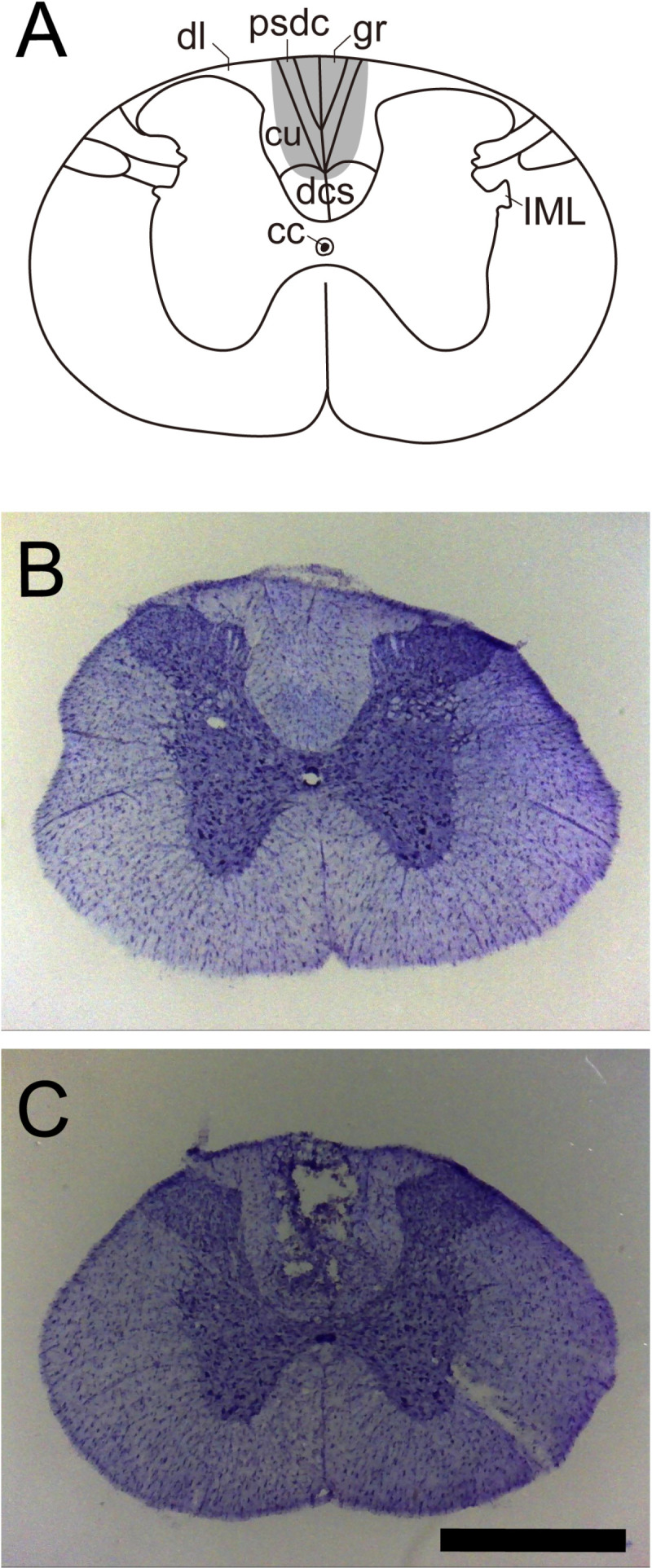
Coronal sections and schematic drawings of a representative DCL at T2. (A) Schematic representation of the thoracic lesion (gray area) according to the spinal cord (T2) atlas by Sengul *et al*. [53]; (B) Intact spinal cord (sham); (C) DCL of the spinal cord. Scale bar, 1 mm. In DCL rats, the ascending sensory fibers in the dorsal columns were damaged with minimal damage to the corticospinal tract. cc: central canal; cu: cuneate fasciculus; dcs: dorsal corticospinal tract; dl: dorsolateral fasciculus; IML: intermediolateral column; psdc: postsynaptic dorsal column pathway.

Histological examination of the spinal cord revealed that the lesion successfully interrupted the dorsal column, being restricted to the dorsomedial portion of the white matter with minimal damage to the dorsal corticospinal tract and intact dorsal horns (Fig 1A, C). Sham animals did not show any evidence of damage to the spinal cord (Fig 1B).

### Effect of DCL on 50-kHz USVs

**Fig 2:**
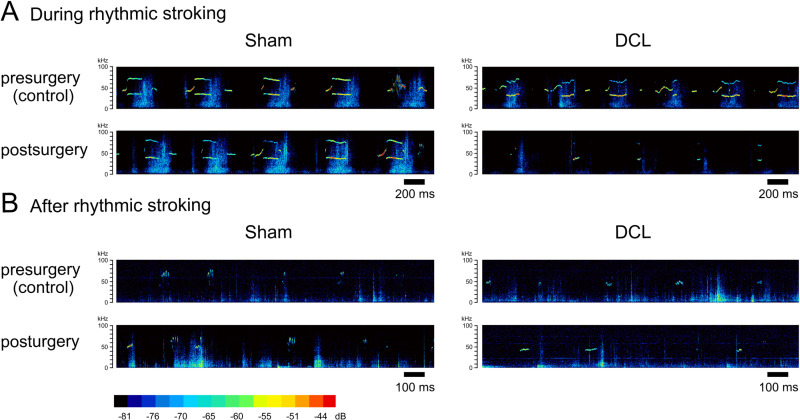
Representative spectrograms of 50-kHz USVs during and after rhythmic stroking in two rats pre- and post-DCL or sham surgery. (A) 50-kHz USVs during rhythmic stroking before surgery. (B) 50-kHz USVs after rhythmic stroking after surgery.

**Fig 3:**
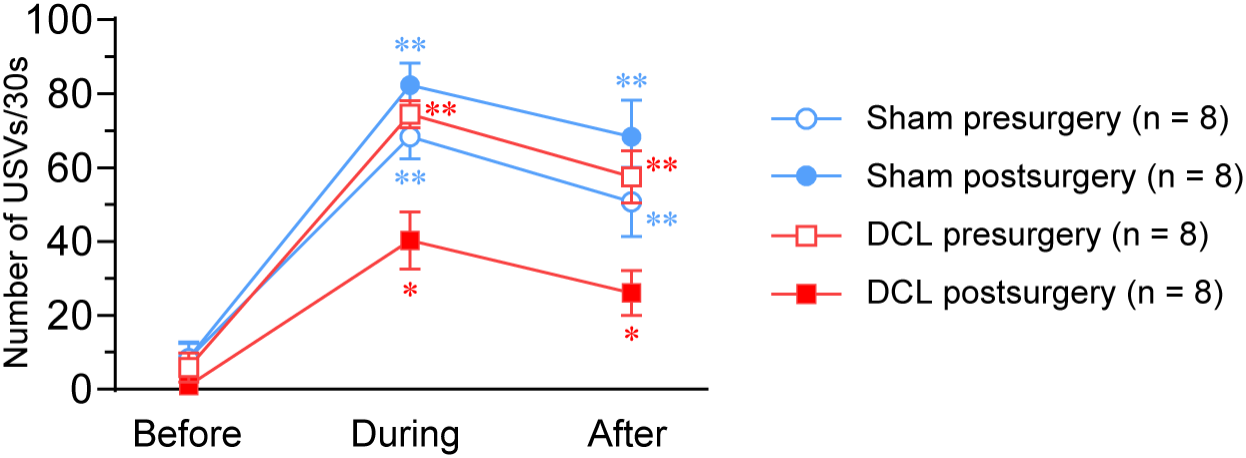
Effects of a DCL on the number of 50-kHz USVs induced by rhythmic stroking. The DCL decreased the number of 50-kHz USVs during and after stroking. * p <  0.01, **p <  0.001 compared to before stroking (baseline). Group means were compared by RM-ANOVA followed by post hoc Bonferroni tests for pair-wise comparisons. N =  8 rats/group.

Fig 2 shows representative sonograms of 50-kHz USVs pre- (control) and postoperative in sham and DCL groups during and after rhythmic stroking. Three-way ANOVA revealed a significant between-group difference in the number of 50-kHz USVs (F [1,28] =  5.823, p =  0.030); significant main effects of treatment (F [1,28] =  4.956, p =  0.043) and time (F [2,28] =  88.022, p <  0.001); and a significant treatment ×  group interaction (F [1,28] =  34.199, p <  0.001), whereas no significant time ×  group interaction was detected (F [2,28] =  1.221, p =  0.299) (Fig 3). In the DCL group, two-way ANOVA revealed significant main effects of treatment (F [1,14] =  49.863, p <  0.001) and time (F [1,14] =  34.224, p <  0.001) and a significant treatment ×  time interaction (F [1,14] =  6.179, p =  0.012). In the sham group, no significant main effect of treatment (F [1,14] =  4.872, p =  0.063) was noted, but a significant main effect of time (F [1,14] =  55.22, p <  0.001) and significant treatment ×  time interaction (F [1,14] =  8.022, p =  0.005) were observed. Two-way ANOVA revealed no significant between-group difference in the number of 50-kHz USVs before surgery (F [1,14] =  0.323, p =  0.579), but a significant between-group difference in the number of 50-kHz USVs was detected after surgery (F [1,14] =  22.345, p <  0.001). One-way ANOVA revealed a significant main effect of time in sham and DCL groups (sham/presurgery, F [2,7] =  57.678, p <  0.001; sham/postsurgery, F [2,7] =  63.960, p <  0.001; DCL/presurgery, F [2,7] =  36.143, p <  0.001; DCL/postsurgery, F [2,7] =  16.857, p <  0.001). Post hoc comparisons showed that the number of 50-kHz USVs during and after rhythmic stroking was significantly higher compared with baseline in both groups.

### Effect of DCL on categorized 50-kHz USVs

**Fig 4:**
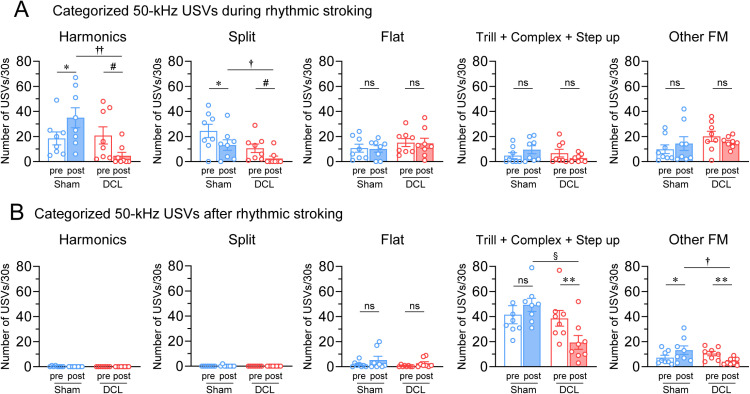
Effects of the DCL on the number of categorized 50-kHz USVs induced by rhythmic stroking. Number of categorized 50-kHz USVs during (A) and after (B) rhythmic stroking in pre- and post-DCL/sham rats. The DCL decreased the number of total 50-kHz USVs during and after stroking. * p <  0.05, **p <  0.01 compared to the presurgery control using paired t-test. ^#^p <  0.05 compared to the presurgery control using Wilcoxon Signed Rank test. ^†^p <  0.05, ^††^p <  0.01 compared to the sham group using Mann–Whitney U test. ^§^p <  0.001 compared to the sham group using unpaired t-test. N =  8 rats/group.

No trill with jump calls or 22-kHz calls were detected in this study. As shown in Fig 4A and B, no 50-kHz subtypes displayed significant differences versus the presurgical control values between the sham and DCL groups (p >  0.05). DCL rats exhibited significant decreases in specific subtypes compared with the presurgical values. Specifically, harmonics (presurgery; 20.9 ±  6.9 calls, postsurgery; 4.6 ±  2.8 calls, z =  − 2.521, p =  0.012) and split calls (presurgery; 10.6 ±  3.4 calls, postsurgery; 2.5 ±  1.9 calls, z =  − 2.173, p =  0.030) significantly decreased during rhythmic stroking (Fig 4A). Hedonic FM calls (presurgery; 38.6 ±  6.3 calls, postsurgery; 19.4 ±  5.5 calls, t =  4.713, p =  0.002) and other FM calls (presurgery; 10.5 ±  1.7 calls, postsurgery; 4.0 ±  1.0 calls, t =  4.351, p =  0.003) significantly decreased after rhythmic stroking (Fig 4B). Compared with the findings in sham rats, DCL rats displayed significant decreases in the number of harmonics (U =  60, p =  0.002) and split calls (U =  53, p =  0.028) during stroking and hedonic FM calls (t =  3.956, p =  0.001) and other FM calls (U =  55, p =  0.015) after stroking. Conversely, sham rats exhibited a significant increase in the number of harmonics (presurgery; 18.5 ±  5.2 calls, postsurgery; 35.0 ±  7.9 calls, t =  − 3.175, p =  0.016) during stroking and other FM calls (presurgery; 7.3 ±  1.9 calls, postsurgery; 13.3 ±  3.4 calls, t =  − 2.542, p =  0.039) after stroking compared with presurgery control. Flat calls during and after stroking did not significantly differ between pre- and postsurgery in both groups (during stroking; sham/presurgery; 10.8 ±  3.2 calls, sham/postsurgery; 10.1 ±  2.6 calls, t =  0.249, p =  0.81; DCL/presurgery; 15.1 ±  3.2 calls, DCL/postsurgery; 14.6 ±  4.0 calls, t =  0.000, p =  1.000; after stroking; sham/presurgery; 1.8 ±  0.8 calls, sham/postsurgery; 5.4 ±  2.9 calls, z =  1.378, p =  0.168, DCL/presurgery; 0.5 ±  0.3 calls, DCL/postsurgery; 2.8 ±  1.3 calls, z =  1.160, p =  0.246, respectively).

### Effect of DCL on behavior

**Fig 5:**
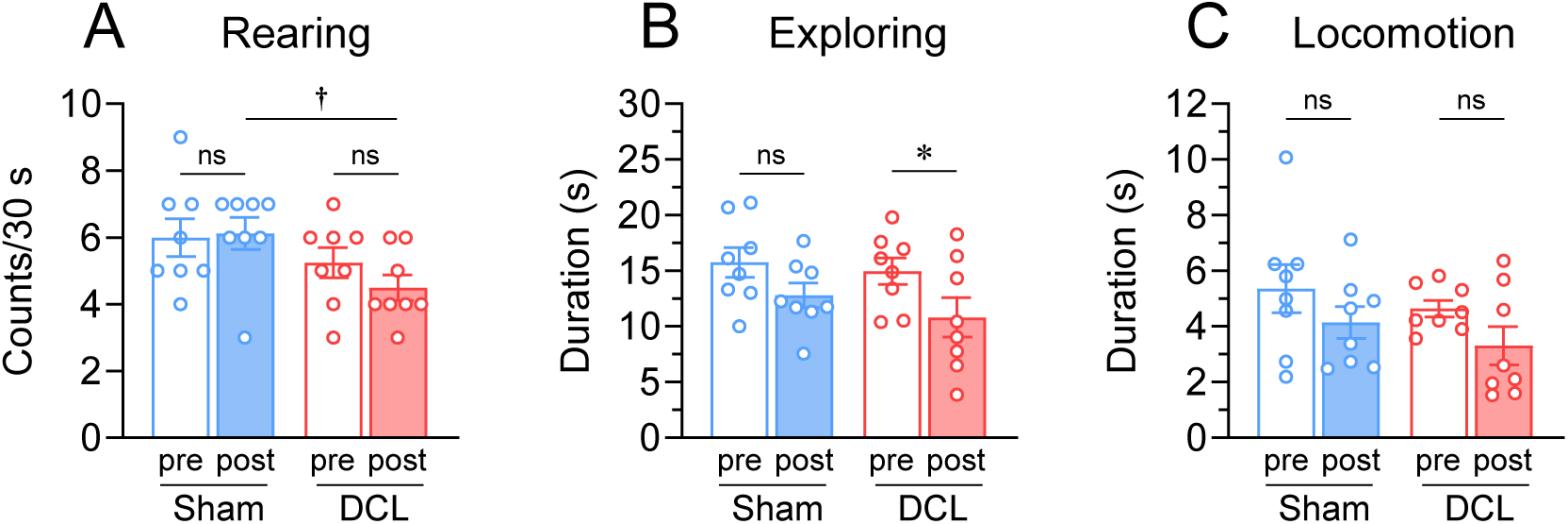
Effects of the DCL on the behavioral activities. (A) Rearing counts. (B) Exploring duration. (C) Locomotion duration. * p <  0.05; significant difference based on paired t-test. ^†^p <  0.05; significant difference based on Mann–Whitney U test. N =  8 rats/group.

After the lesion of the dorsal column at T2, functional deficits in the hindpaw, with foot-slips during rearing, were observed. This was not the case in sham-operated rats. No immobilization or freezing was observed in either group. Sham rats displayed no significant difference in behaviors between before and after surgery (rearing/ presurgery; 6.0 ±  0.6 counts, postsurgery; 6.1 ±  0.5 counts, z =  0.171, p =  0.865; exploring/ presurgery; 15.8 ±  1.4 s, postsurgery; 12.8 ±  1.1 s, t =  2.297, p =  0.055; locomotion/ presurgery; 5.4 ±  0.9 s, postsurgery; 4.1 ±  0.6 s, t =  1.401, p =  0.204; Fig 5A–C). Compared with the presurgical control values, DCL rats exhibited a significant decrease in exploring (presurgery; 15.0 ±  1.2 s, postsurgery; 10.8 ±  1.8 s, t =  2.458, p =  0.044) but not in rearing (presurgery; 5.3 ±  0.5 counts, postsurgery; 4.5 ±  0.4 counts, t =  1.337, p =  0.218) and locomotion (presurgery; 4.6 ±  0.3 s, postsurgery; 3.3 ±  0.7 counts, t =  2.026, p =  0.082). Compared with the results in sham rats, postoperative DCL rats displayed a significant decrease in rearing (U =  54.5, p =  0.012) but not in exploring (t =  0.954, p =  0.356) and locomotion (t =  0.928, p =  0.369).

### Approach latency

**Fig 6:**
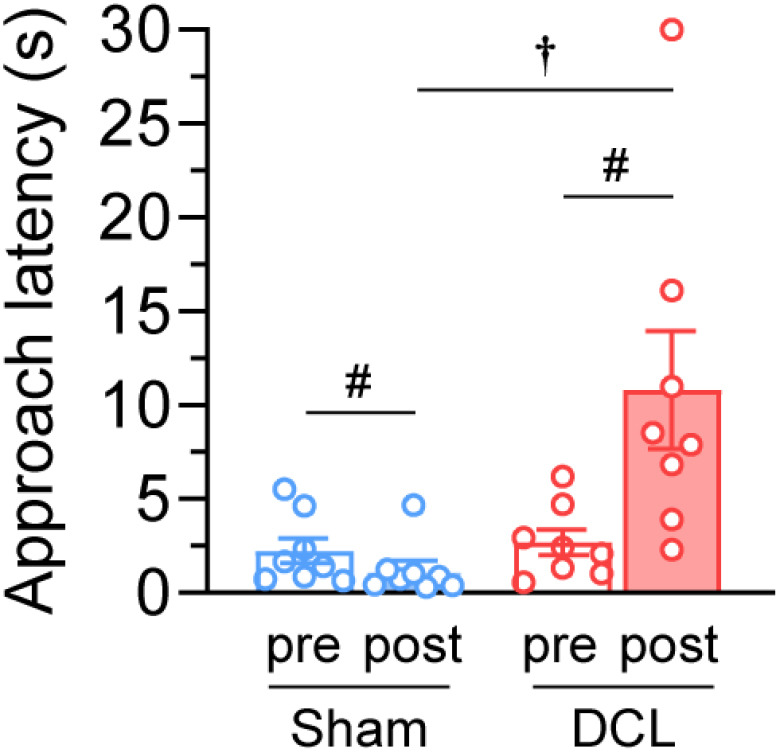
Effects of the DCL on approach latency. ^#^p <  0.05; significant difference based on the Wilcoxon signed rank test. ^†^p <  0.05; significant difference based on Mann–Whitney U test. N =  8 rats/group.

DCL rats exhibited a significant increase in approach latency compared with the presurgical control findings (presurgery; 2.7 ±  0.7 s, postsurgery; 10.8 ±  3.1 s, z =  2.521, p =  0.012; Fig 6). Sham rats displayed a significant decrease in approach latency compared with the presurgical control (presurgery; 2.2 ±  0.7 s, postsurgery; 1.2 ±  0.5 s, z =  − 1.96, p =  0.05). Preoperative DCL rats exhibited no difference compared with sham rats in approach latency (U =  26.0, p =  0.574) but postoperative DCL rats showed a significant increase in approach latency (U =  9.0, p =  0.016).

### Mechanical sensory thresholds

**Fig 7:**
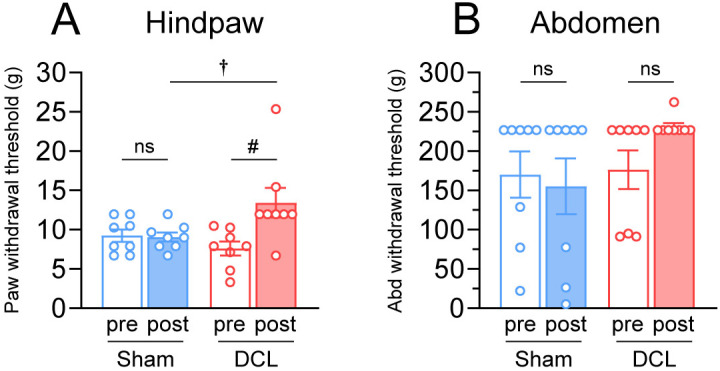
Effects of DCL on mechanical sensory thresholds. Tactile thresholds were determined by probing the plantar aspect of the hindpaw or abdomen with von Frey filaments by the “up-down” method (see text for details). The bilateral DCL resulted in an increase of hindpaw withdrawal thresholds but not in the abdomen. In the sham groups, the hindpaw and abdomen withdrawal thresholds did not change from preoperative values. ^#^p <  0.05; significant difference based on the Wilcoxon signed rank test. ^†^p <  0.05; significant difference based on Mann–Whitney U test. N =  8 rats/group.

DCL rats showed a significant increase in mechanical sensory thresholds in the hindpaw compared with presurgical control (presurgery; 7.6 ±  0.9 g, postsurgery; 13.4 ±  1.9 g, z =  2.521, p =  0.012; Fig 7A), but not in the abdomen (presurgery; 176.5 ±  24.6 g, postsurgery; 231.3 ±  4.5 g, z =  1.604, p =  0.109; Fig 7B). Sham rats showed no significant changes before and after surgery in hindpaw (presurgery; 9.3 ±  0.8 g, postsurgery; 9.1 ±  0.6 g, t =  0.251, p =  0.809; Fig 7A) and abdomen (presurgery; 170.3 ±  29.4 g, postsurgery; 155.5 ±  35.5 g, z =  − 0.535, p =  0.593; Fig 7B).

## Discussion

In this study, we investigated whether the somatosensory signals conveyed by the dorsal column are involved in positive 50-kHz USVs and reward-related behaviors induced by rhythmic stroking. DCL decreased the number of 50-kHz and motivated behaviors. The results of this study suggested that the dorsal column provides critical sensory signals involved in inducing positive emotion and behaviors in response to rhythmic stroking. Our results supported the findings in humans that mechanoreceptive A-fiber signals, which are believed to be mainly transmitted by the dorsal column tract, are necessary to perceive the intensity and pleasantness of gentle stroking [41].

Surgical injury of the dorsal column at the upper thoracic level allowed us to interrupt a major portion of the somatosensory information transmitted from the lower trunk to the brain, leaving the corticospinal tract intact. The histological verification revealed that the dorsal corticospinal tract was largely spared using our technique. In addition, behavioral activities regarding locomotion and rearing were not noticeably different in either the sham or DCL group between before and after surgery. This result is consistent with previous studies illustrated that thoracic DCLs led no or few motor deficits [60–63]. Thus, in this study, we minimized the impact on motor function and examined the effect of destroying the target area, namely the ascending somatosensory pathway, on the 50-kHz USVs.

Consistent with our previous research, the present study showed that most common subtypes observed during rhythmic stroking were harmonics and split calls, while after rhythmic stroking the most frequent were hedonic FM (trill, complex, and step up) calls [28,35]. DCL rats reduced 50-kHz USVs, with almost completely absent harmonics and split calls during rhythmic stroking and nearly half hedonic FM calls after rhythmic stroking. The present results supported the idea that harmonics and split calls during rhythmic stroking and FM calls after rhythmic stroking indicated the positive affective state in rats [28,35,42].

Consistent with our previous research, the present study found that rhythmic stroking can promote approach behavior in sham-operated rats [28]. Contrarily, DCL surgery significantly increased approach latencies. Approach behavior is a highly motivated reward-seeking behavior and a reliable marker of positive emotion in rats [21,23,24,28]. Therefore, it is suggested that DCL surgery effectively reduced the positive valence of rhythmic stroking and decreased reward-related approach behavior.

Consistent with our previous research, innate active behaviors such as locomotion, exploration, and rearing [28,42] were observed after rhythmic stroking. DCL decreased rearing and exploring. It is suggested that the dorsal column partly provides afferent mechanisms related to incentive-motivated exploration. Further studies are needed to elucidate the neural mechanisms underlying motivated exploratory behaviors induced by tactile reward [28,35,42,64].

In this study, the von Frey test showed increased mechanical sensory thresholds in the hindpaw, but not in the abdomen, after the thoracic DCL. Previous studies reported that animals with large thoracic lesions (whole spinal cord contusion or hemisection) suffer allodynia in the hindpaw [65,66]. In contrast, a small midline lesion (limited to gracile fasciculus) of the thoracic dorsal column did not affect the mechanical threshold in the hindpaw [67]. In this study, the dorsal horn was preserved, but the gracile and cuneate fasciculi were almost fully destroyed, suggesting that destruction of the dorsal fasciculus caused the sensory impairment in the hindpaw detected by the von Frey test. As for the abdomen, as some rats did not respond to the maximum value (100 g) at the time of control data collection, the von Frey test to the abdomen could hardly evaluate accurately sensory dysfunction in rats. Nonetheless, the results indicated that our spinal cord injury can detect tactile sensory deficits without causing hyperalgesia using the von Frey test. Further, the sensory dysfunction due to the DCL at the upper thoracic level can be evaluated at the hindpaw.

The dorsal column pathway carries tactile information, especially discriminatory touch, whereas the ventral spinothalamic tract transmit crude touch and pressure information in rats [50]. In this study, DCL decreased the 50-kHz USVs and motivated behaviors that code the positive affective valence of somatosensory stimulation. Therefore, it is suggested that discriminative touch information is involved in motivational affective responses. The notion is supported by the results of human and animal studies in which stroking (discriminative touch) more effectively generated affective emotion than light touch (crude touch) [28,40,42].

The dorsal column tract projects to the dorsal column nuclei, which project targets throughout the brain including the ascending reticular activating system [68], and the ventral tegmental area dopamine neurons receive excitatory input from the diverse brainstem nuclei, which are the sites of projection from the dorsal column nuclei [69–71]. We previously reported that the mesoaccumbal dopamine system is involved in the emission of 50-kHz USVs during rhythmic stroking, which induced motivated behaviors [28,35,42]. Therefore, it is suggested that the areas in the brain activated by rhythmic stroking are involved in reward and arousal via somatosensory signals conveyed by the dorsal column, thereby triggering active behaviors. Further studies are needed to clarify the precise supraspinal structures involved in the skin-to-brain circuit regulating positive emotion and motivation induced by tactile stimulation.

In this study, we used only male rats because our previous experiments examining rat USVs induced by tactile stimuli had been conducted in males [27,28,35,42]. Although using female rats is important, designing a more intricate experiment that accounts for the effects of the ovarian cycle—known to influence functional activity in the brain reward system and emotion-related behaviors—was beyond the scope of this study [72–74].

## Conclusions

In summary, the dorsal column plays a key role as an ascending pathway to evoke hedonic 50-kHz USVs induced by tactile reward in rats. Our animal model using rhythmic stroking may contribute to clarify how tactile information involved in the generation of positive 50-kH USVs is processed and conveyed from the skin to the brain. Our results serve as a basis for the neural mechanisms underlying the positive affective state induced by massage and tactile care using touch in the treatment of mental disorders, such as anxiety and depression.
